# A Non-Invasive Integrated Model for Accurate Preoperative Identification of the Aggressive Macrotrabecular-Massive Subtype of Hepatocellular Carcinoma: A Single-Center Retrospective Study

**DOI:** 10.3390/diagnostics16060877

**Published:** 2026-03-16

**Authors:** Yuanqing Zhang, Yang He, Yifei Chen, Xiaorong Lv, Rong Yang, Guo Chen, Fang Nie

**Affiliations:** 1 Ultrasound Medicine Center, The Second Hospital of Lanzhou University, Lanzhou 730030, China; yuanqinzh@163.com (Y.Z.); lxr20010729@163.com (X.L.); yangr8915@163.com (R.Y.); m15293179685@163.com (G.C.); 2 Ultrasound Center, Gansu Provincial Clinical Research Center for Ultrasound Medicine, Lanzhou 730030, China; 3 Intelligent Ultrasound Center, Gansu Provincial Engineering Research Center for Intelligent Ultrasound Medicine, Lanzhou 730030, China; 4 Lanzhou University, Lanzhou 730030, China; 18909357625@163.com (Y.H.); 17750853099@163.com (Y.C.)

**Keywords:** liver, macrotrabecular-massive hepatocellular carcinoma, contrast-enhanced ultrasound, preoperative prediction, diagnosis

## Abstract

**Objective:** The objective of this study was to develop and validate a predictive model for MTM-HCC by integrating preoperative ultrasound (US) and contrast-enhanced ultrasound (CEUS) features with relevant clinical characteristics. **Methods:** This retrospective study analyzed data from patients with histopathologically confirmed hepatocellular carcinoma who underwent preoperative CEUS examination at the Ultrasound Department of the Lanzhou University Second Hospital between December 2021 and March 2025. The study cohort comprised 45 patients diagnosed with MTM-HCC and 194 patients with non-MTM-HCC. Ultrasound and CEUS images were independently reviewed by two senior abdominal radiologists with extensive experience in hepatic imaging, ensuring objective feature assessment. Clinical variables and imaging characteristics were systematically compared between the two groups to identify distinguishing patterns. To evaluate the associations among clinical data, ultrasound-derived features, and MTM-HCC, univariate analyses were first performed, followed by multivariate logistic regression to construct and assess predictive models. **Results:** A total of 239 patients (mean age: 57.28 ± 9.60 years; 187 males and 52 females) were included in the analysis. Among them, 45 HCC patients (18.8%) were classified as MTM-HCC. Multivariate analysis identified four independent predictors: elevated alpha-fetoprotein (AFP ≥ 467 ng/mL) (OR = 8.5, 95% CI: 4.2–17.30; *p* < 0.001), presence of non-enhancing necrotic areas (OR = 5.92, 95% CI: 1.82–19.30, *p* = 0.003), intratumoral arteries (OR = 6.61, 95% CI: 2.28–19.22, *p* < 0.001), and peritumoral feeding arteries (OR = 3.13, 95% CI: 1.15–8.50, *p* = 0.025). **Conclusions:** An integrated prediction model that combines ultrasound imaging and clinical parameters offers a feasible, non-invasive approach for accurate preoperative identification of MTM-HCC.

## 1. Introduction

Liver cancer remains a major global health burden, ranking as the third leading cause of cancer-related mortality worldwide [1,2]. The burden is especially heavy in East Asia, where nearly half of all global liver cancer cases and deaths occur in China [2]. Among primary liver cancers, HCC accounts for the most significant proportion and is the most common pathological type encountered in clinical practice [3,4]. Chronic infection with hepatitis B virus (HBV) or hepatitis C virus (HCV) significantly elevates HCC risk, primarily through sustained inflammation and fibrogenesis, which promote carcinogenesis [1]. In addition to viral causes, long-term alcohol consumption accelerates liver damage and increases the likelihood of cirrhosis and HCC, especially at high intake levels, and nonalcoholic steatohepatitis (NASH)—the progressive form of nonalcoholic fatty liver disease associated with obesity and metabolic syndrome—has emerged as a significant risk factor for HCC development [5]. The development of HCC is closely linked to chronic liver injury and a spectrum of established risk factors. Liver cirrhosis, regardless of etiology, remains the most important premalignant condition [6]. The high heterogeneity of HCC at genomic and histopathological levels limits the effectiveness of treatment strategies based solely on clinical staging systems such as the Barcelona Clinic Liver Cancer (BCLC) [7]. The World Health Organization (WHO) Classification of Tumours categorizes HCC into eight distinct pathological subtypes [8]. Among the recognized histological subtypes of HCC, the macrotrabecular-massive (MTM) variant has attracted particular attention because of its distinctly aggressive biological and molecular profile. This subtype is characterized by marked angiogenesis, frequent vascular invasion, and activation of proliferative signaling pathways, features that collectively underpin its unfavorable clinical course [9]. At present, definitive diagnosis of MTM-HCC still depends on postoperative histopathological assessment of resected tumor specimens [10], which limits timely risk stratification and treatment planning in the preoperative setting [11]. Histopathologically, MTM-HCC demonstrates characteristic microscopic features, predominantly exhibiting a macrotrabecular growth pattern. The tumor trabeculae measure more than six cell layers in thickness and are composed of densely packed tumor cell cords. These structures are circumscribed by vascular channels lined with endothelial cells. Moreover, the abundant presence of CD34-positive vascular components in MTM-HCC collectively contributes to the formation of its distinctive “vessels that encapsulate tumor clusters” (VETC) pattern [12,13]. This histological feature is closely associated with rapid tumor spread, early recurrence, and poor prognosis [11,14]. Accurate preoperative identification of MTM-HCC is of great clinical value for formulating individualized treatment plans and improving patient prognosis. At present, studies on the imaging features of MTM-HCC are still scarce, and there is no consensus on the optimal predictive biomarkers for this subtype. Among existing imaging studies, Mulé et al. [15] reported that extensive tumor necrosis observed in contrast-enhanced magnetic resonance imaging (MRI) is an independent predictive factor for MTM-HCC, which has high specificity in identifying this subtype. Feng et al. [12] confirmed through computed tomography (CT) imaging studies that intratumoral hemorrhage and intratumoral necrosis can serve as independent predictive indicators for MTM-HCC. While magnetic resonance imaging (MRI) and computed tomography (CT) have demonstrated significant diagnostic value for MTM-HCC, they remain constrained by costs and accessibility. More importantly, CEUS offers superior real-time dynamic imaging capabilities, enabling the capture of ultra-early microvascular enhancement features, such as intratumoral arteries, which may be transient or overlooked during the fixed-time phase acquisitions of CT or MRI. This unique advantage positions CEUS as a highly sensitive and accessible tool for the preoperative identification of tumor aggressiveness. Beyond lesion detection, modern US techniques—particularly Doppler imaging and CEUS—allow assessment of tissue perfusion, vascular architecture, and hemodynamic behavior, thereby providing functional information closely related to disease biology [16,17,18]. This real-time capability facilitates a more direct evaluation of tumor microvascular architecture and hemodynamic behavior [19]. For example, Wilson et al. demonstrated that CEUS could accurately characterize focal liver lesions by capturing dynamic vascular patterns, achieving diagnostic performance comparable to cross-sectional imaging [20]. Collectively, these studies suggest that US is not merely a screening or descriptive tool, but a robust imaging modality capable of supporting preoperative prediction and clinical decision-making. However, systematic studies on the US features of MTM-HCC remain relatively scarce, and the value of CEUS in the preoperative diagnosis of this subtype has not been fully explored [21,22]. Therefore, an urgent need exists to systematically evaluate the potential of preoperative US features combined with clinical indicators of MTM-HCC, and provide a new non-invasive diagnostic approach for the early identification of this aggressive subtype. Accordingly, this study aimed to evaluate the value of ultrasound-based features combined with clinical parameters for the preoperative identification of MTM-HCC.

## 2. Materials and Methods

### 2.1. General Information

Consecutive patients with HCC who underwent preoperative conventional US and CEUS examinations using a Resona R9 ultrasound system (Mindray, Shenzhen, China) in the Department of US at the Second Hospital of Lanzhou University between December 2021 and March 2025 were initially identified. The diagnosis of HCC was histopathologically confirmed in all included patients by either surgical resection or biopsy.

**Figure 1:**
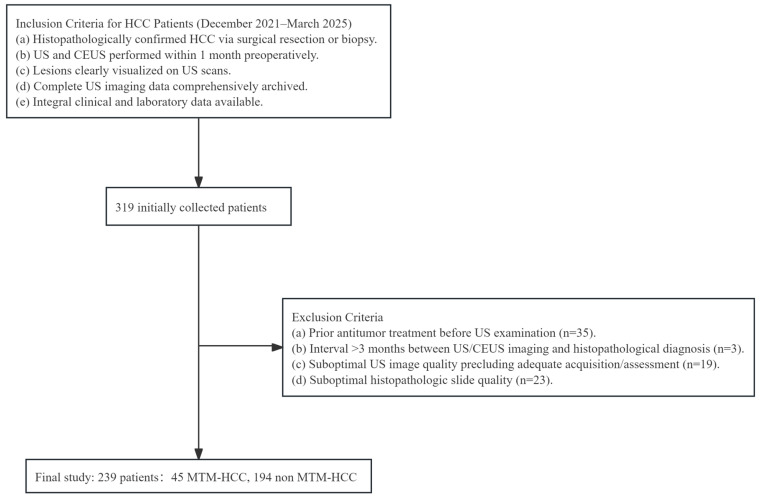
Flow diagram for patient enrollment in this study. US: conventional ultrasound; CEUS: contrast-enhanced ultrasound; HCC: hepatocellular carcinoma; MTM-HCC: thick trabecular-mass-type hepatocellular carcinoma.

For the cohort, consecutive patients with HCC confirmed by pathology who underwent preoperative conventional US and CEUS at our institution were retrospectively reviewed. Patients were enrolled if they met the following criteria: (1) conventional US and liver CEUS using SonoVue^®^ (Bracco, Milan, Italy) were performed within 1 month before surgery, (2) the diagnosis of HCC was confirmed by postoperative pathological examination, and (3) no antitumor treatment was administered prior to US examination. The exclusion criteria were as follows: (1) poor-quality US or CEUS images, including incomplete arterial, portal venous, or late phase acquisition, missed lesions, or excessive respiratory motion; (2) an interval of more than 3 months between US examination and pathological diagnosis; and (3) incomplete clinical, laboratory, or pathological data. After applying these criteria, a total of 239 patients were included in the final study cohort. A flowchart of patient enrollment and exclusion is shown in Figure 1.

### 2.2. Examination Method

CEUS was performed using a Philips EPIQ7 ultrasound system (Philips Healthcare, Amsterdam, The Netherlands) with a C5–1 convex transducer (1–5 MHz) under a low-mechanical-index setting. Following identification of the target lesion, CEUS was obtained; in patients with multiple lesions, the largest lesion and its maximal cross-sectional plane were selected for evaluation. SonoVue was administered as a 2.4 mL bolus via the antecubital vein, immediately followed by a 5 mL saline flush. CEUS cine loops were acquired continuously in real time and digitally stored for subsequent analysis. All examinations were independently interpreted by two radiologists with more than 5 years of experience in hepatic CEUS, both of whom were blinded to the pathological findings.

### 2.3. Histopathological Analysis

All histological sections were reviewed by a pathologist with 16 years of experience (W.D. Li), who was blinded to other clinical and imaging findings.

Diagnosis was based on previous pathological criteria: (1) The dominant architectural pattern of the tumor is the macrotrabecular pattern (with trabecular thickness exceeding six cells) [22,23,24]. (2) The Edmondson–Steiner grade and immunohistochemical markers (Ki-67 index, CD34, Glypican-3, and CK19) were recorded simultaneously.

### 2.4. Image Analysis

To ensure the reliability of imaging interpretation, all conventional US and CEUS images were independently reviewed by two US physicians (with 15 and 13 years of experience in hepatic US, respectively) who were blinded to the patients’ clinical and pathological information. In cases of disagreement, a consensus was reached through joint discussion. For patients with multiple lesions, the largest tumor was selected for evaluation. According to the American College of Radiology CEUS Liver Imaging Reporting and Data System (LI-RADS, version 2017), LI-RADS categories were assigned in 181 of the 239 patients.

The following US and CEUS characteristics were evaluated for each lesion: (1) number of lesions, (2) maximum tumor diameter, (3) shape, (4) echogenicity, (5) echo homogeneity, (6) tumor margin, (7) tumor capsule, (8) nodule-in-nodule appearance, (9) peritumoral blood flow signal graded according to the Adler classification, (10) arterial phase (AP) enhancement onset time, (11) AP enhancement degree, (12) expansion of the enhanced area in the AP, (13) AP enhancement pattern, (14) washout onset time, (15) portal venous phase (PVP) washout degree, (16) delayed phase clearance pattern, (17) intratumoral necrosis, (18) intratumoral artery, (19) peritumoral feeding artery, and (20) portal vein tumor thrombus.

### 2.5. Statistical Analysis

Variables associated with MTM-HCC were first screened using univariate analysis and subsequently underwent multivariable binary logistic regression to identify independent predictors. Serum alpha-fetoprotein (AFP) was initially analyzed as a continuous variable in both the univariate and multivariable models. For construction of the final combined predictive model, AFP was dichotomized to improve clinical interpretability, with the optimal cutoff determined by receiver operating characteristic (ROC) curve analysis using the Youden index. The dichotomized AFP variable was then incorporated into the model together with selected CEUS features.

ROC curves were used to assess the discriminative performance of the model, and diagnostic efficacy was evaluated based on sensitivity, specificity, accuracy, positive predictive value, negative predictive value, and area under the ROC curve (AUC).

Statistical analyses were performed using SPSS 26.0 (IBM, Armonk, NY, USA) and R 4.4.2 (https://www.r-project.org/, accessed on 15 October 2025). Measurement data with a normal distribution were expressed as $\bar{x}$ ± s, and comparisons between groups were conducted using the independent samples *t*-test. Data with a non-normal distribution were presented as M (Q1, Q3), and intergroup comparisons were performed using the Mann–Whitney U test. Categorical variables were expressed as frequencies or percentages, and differences between groups were tested using the χ^2^ test. A *p*-value < 0.05 was considered statistically significant.

### 2.6. Sample Size Determination

As this was a retrospective study, no formal a priori sample size calculation was performed. The adequacy of the sample size for multivariable logistic regression was evaluated using the events-per-variable (EPV) principle. According to widely accepted recommendations, at least 10 outcome events are required per predictor variable to ensure model stability. In the present cohort, 239 patients were included, among whom 45 were diagnosed with MTM-HCC. Based on this number of events, the dataset could reliably support approximately four to five predictor variables in the multivariable logistic regression model. The final model included four predictors, satisfying the EPV ≥ 10 criterion and indicating that the sample size was sufficient for model development.

## 3. Results

### 3.1. Clinical and Pathological Baseline Data of Patients

A total of 239 patients were included in the analysis, comprising 194 non-MTM-HCC and 45 MTM-HCC patients. The two groups were comparable in terms of sex distribution, with no significant difference observed (*p* = 0.751). In contrast, patients with MTM-HCC exhibited markedly elevated serum alpha-fetoprotein (AFP) levels compared with those in the non-MTM-HCC group (median, 1210.0 vs. 32.6 ng/mL; *p* < 0.001). Regarding liver function-related parameters, aspartate aminotransferase (AST) levels were significantly higher in the MTM-HCC group (*p* = 0.005), whereas no significant differences were observed in alanine aminotransferase (ALT), albumin (ALB), total bilirubin (TBil), hemoglobin (Hb), or platelet counts (PLT) between the two groups. In terms of inflammatory status, patients with MTM-HCC showed higher peripheral neutrophil (NEU) counts (*p* = 0.045) and a significantly increased neutrophil-to-lymphocyte ratio (NLR) (*p* = 0.009), indicating a more pronounced inflammatory response. No significant differences were observed between the two groups in terms of hepatitis B virus (HBV) infection status or the prevalence of liver cirrhosis. From a pathological perspective, MTM-HCC was more frequently associated with CK19+ (33.3% vs. 17.0%, *p* = 0.014), higher Edmondson–Steiner grades (III–IV) (33.3% vs. 11.3%, *p* < 0.001), and a significantly higher Ki67 index (median, 40% vs. 30%; *p* < 0.001). In contrast, no significant differences were observed in the expression of CD34 or glypican-3 (Table 1).

### 3.2. Imaging Findings of MTM-HCC on US and CEUS

**Figure 2:**
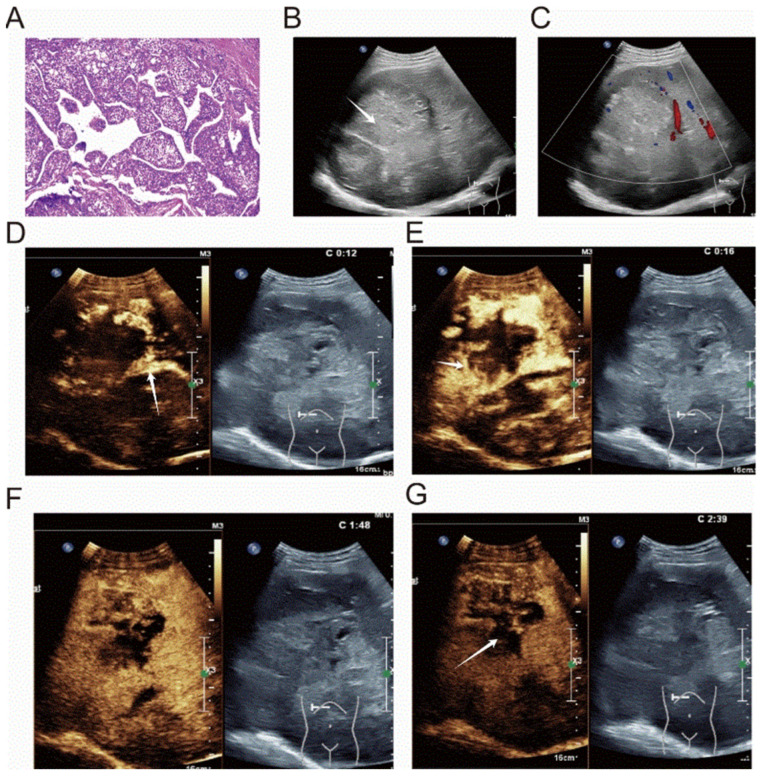
Diagnostic images for a 60-year-old male with hepatitis B virus-related cirrhosis and macrotrabecular-massive hepatocellular carcinoma (MTM-HCC). The laboratory data showed an AFP level of 1210 ng/mL, AST of 65 U/L, and neutrophil-to-lymphocyte ratio (NLR) > 6. Pathological immunohistochemistry demonstrated a Ki-67 index of 60%. (**A**) The micrograph reveals a macrotrabecular-massive growth pattern. (**B**) The two-dimensional ultrasound image demonstrates an approximately 8.7 cm HCC in segment VIII of the right hepatic lobe. The mass exhibits heterogeneous echogenicity, a peripheral hypoechoic halo, and a “nodule-in-nodule” appearance. The lesion is indicated by white arrows. (**C**) Peripheral rim-like blood flow signals are observed around the mass. (**D**–**G**): Preoperative contrast-enhanced US (CEUS) images. (**D**,**E**): In the arterial phase, the lesion shows peripheral nodular hyperenhancement. Intratumoral arteries (**D**) and peritumoral arteries (**E**) are visible, each marked by white arrows. (**F**) In the portal venous phase, the lesion exhibits marked washout, appearing hypoechoic. (**G**) In the delayed phase, the lesion shows sustained hypoenhancement. Non-enhancing necrotic areas are present throughout all phases. According to the CEUS Liver Imaging Reporting and Data System (CEUS LI-RADS) 2017, this nodule was classified as LR-M.

Compared with non-MTM-HCC lesions, MTM-HCC lesions were significantly larger, with a greater maximum tumor diameter (median, 6.6 cm vs. 3.7 cm; *p* < 0.001) and a higher proportion of tumors larger than 5 cm (30 of 45 [66.7%] vs. 60 of 194 [30.9%], *p* < 0.001). MTM-HCC more frequently exhibited heterogeneous echogenicity (42 of 45 [93.3%] vs. 141 of 194 [72.7%], *p* = 0.003) and indistinct tumor margins (34 of 45 [75.6%] vs. 83 of 194 [42.8%]), *p* < 0.001). The nodule-in-nodule sign was more common in MTM-HCC than in non-MTM-HCC (29 of 45 [64.4%] vs. 81 of 194 [41.8%], *p* = 0.006). On CEUS, MTM-HCC demonstrated less frequent arterial-phase expansion of the enhanced area (3/45 [6.7%] vs. 37/194 [19.1%], *p* = 0.045) but significantly more frequent arterial-phase peripheral nodular enhancement (31/45 [68.9%] vs. 49/194 [25.3%], *p* < 0.001). In the portal venous phase (PVP), MTM-HCC exhibited a more pronounced degree of washout compared with non-MTM-HCC (21 of 45 [46.7%] vs. 60 of 194 [30.9%], *p* = 0.027). MTM-HCC more commonly exhibited non-enhancing necrotic areas compared with non-MTM-HCC (30/45 [66.7%] vs. 46/194 [23.7%], *p* < 0.001). Intratumoral arteries were more frequently observed in MTM-HCC (33 of 45 [73.3%] vs. 45 of 194 [23.2%], *p* < 0.001), as were peritumoral feeding arteries (28 of 45 [62.2%] vs. 70 of 194 [36.1%], *p* = 0.001). Moreover, the incidence of venous tumor thrombus was significantly higher in MTM-HCC compared with non-MTM-HCC (21/45 [46.7%] vs. 39/194 [20.1%], *p* < 0.001) (Table 2.). A typical image of MTM-HCC is shown in Figure 2.

### 3.3. Development of a Predictive Model for MTM-HCC

In the multivariable logistic regression analysis, elevated serum AFP (AFP ≥ 467 ng/mL) was associated with the MTM subtype (OR = 8.5, 95% CI: 4.2–17.30; *p* < 0.001). Among the imaging features, non-enhancing necrotic areas (OR = 5.92, 95% CI: 1.82–19.30; *p* = 0.003), intratumoral arteries (OR = 6.61, 95% CI: 2.28–19.22; *p* < 0.001), and the presence of a peritumoral feeding artery (OR = 3.13, 95% CI: 1.15–8.50; *p* = 0.025) were independently associated with MTM-HCC (Table 3).Using these four independent predictors, we constructed a combined predictive model. The diagnostic performance of the model is presented in Table 4. The model achieved an AUC of 0.915, with an accuracy of 0.879(95% CI: 0.830–0.917), sensitivity of 0.887 (95% CI: 0.842–0.931), specificity of 0.844 (95% CI: 0.739–0.950), positive predictive value of 0.961 (95% CI: 0.932–0.989), and negative predictive value of 0.633 (95% CI: 0.511–0.755). The optimal cut-off value was 0.255.

By integrating clinical variables and CEUS features using a multivariable logistic regression approach, a combined prediction model was established for identifying the MTM subtype of HCC (variables: AFP ≥ 467 ng/mL, non-enhanced intratumoral necrosis, intratumoral artery, and peritumoral feeding artery).

**Figure 3:**
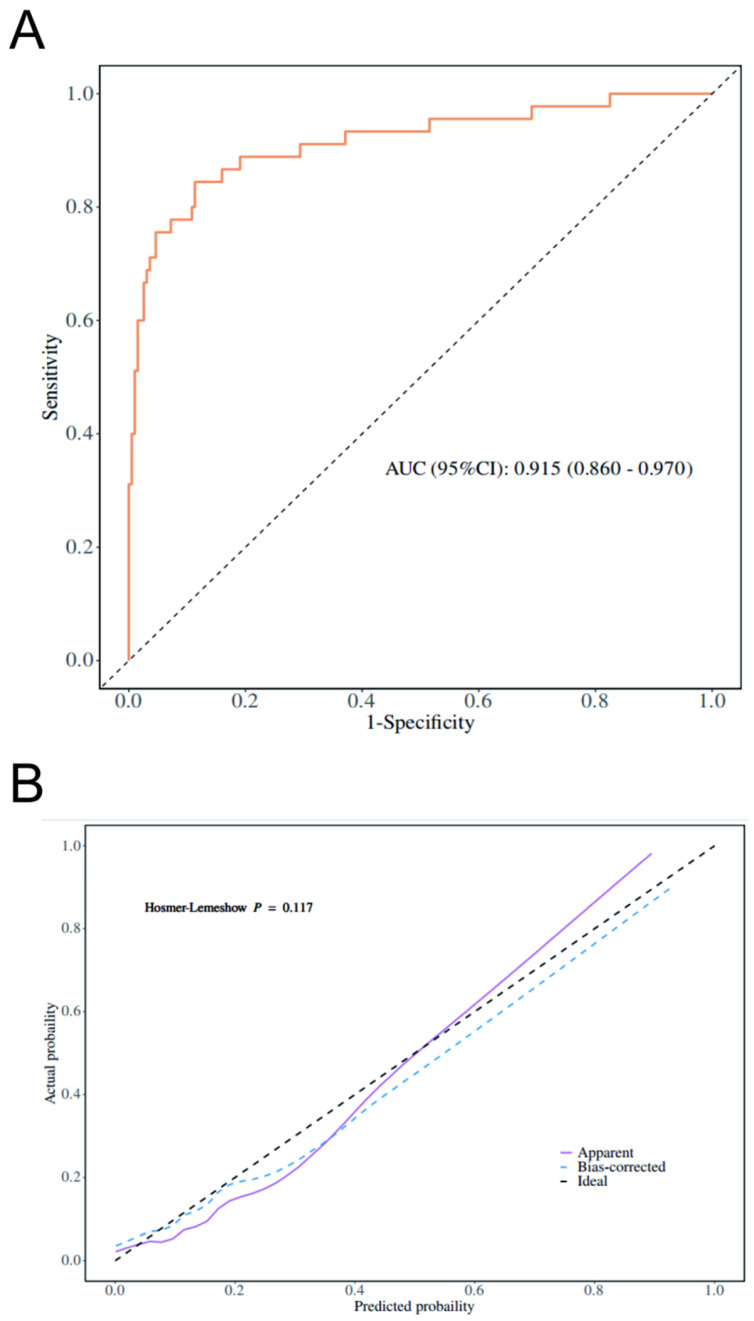
Performance evaluation of the predictive model for macrotrabecular-massive hepatocellular carcinoma (MTM-HCC). (**A**) Receiver operating characteristic (ROC) curve illustrating the model’s discriminatory ability, with an area under the curve (AUC) of 0.915 (95% confidence interval [CI]: 0.860–0.970). (**B**) Calibration plot showing the agreement between predicted and actual probabilities. The black dashed line represents ideal prediction. The Hosmer–Lemeshow test yields a *p*-value of 0.117, indicating good calibration of the model.

Using multivariable logistic regression, we developed a combined model to predict the MTM subtype of HCC. This model integrates three CEUS features and an alpha-fetoprotein (AFP) level ≥ 467 ng/mL. In the study cohort, the model showed strong performance, with an area under the curve (AUC) of 0.915 (95% CI: 0.860–0.970; Figure 3A), a sensitivity of 88.7%, and a specificity of 84.4%. The calibration curve indicated close agreement between the predicted risk and the actual outcomes (Figure 3B).

**Figure 4:**
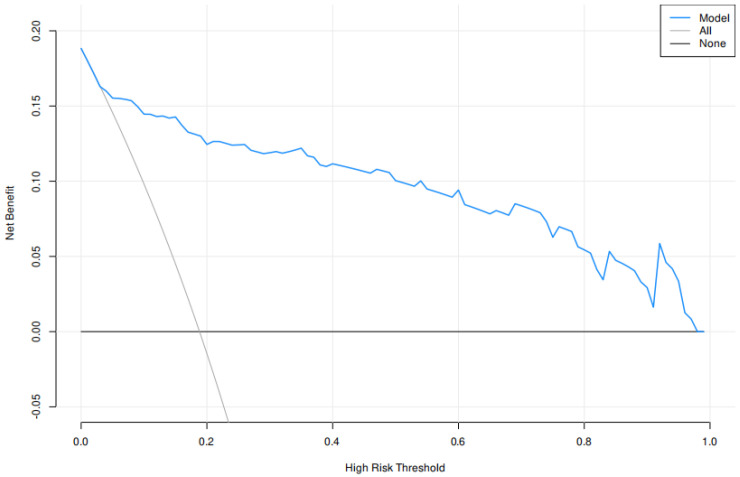
Decision curve analysis for the MTM-HCC predictive model. Note: The y-axis represents net benefit, and the x-axis represents the threshold probability for predicting MTM-HCC. The blue line represents the combined predictive model; the light gray line represents the strategy of treating all patients; and the dark gray line represents the strategy of treating none. The model demonstrates a positive net benefit across a wide range of threshold probabilities (approximately 0.1–0.8), indicating its clinical utility in guiding preoperative decision-making.

Decision curve analysis was performed to evaluate the clinical utility of the predictive model. As shown in Figure 4, the model demonstrated a positive net benefit across a wide range of threshold probabilities, indicating its potential value in clinical decision-making.

## 4. Discussion

This study demonstrates that the combination of serum alpha-fetoprotein (AFP) with specific CEUS features—non-enhanced necrosis within the tumor, intratumoral arteries, and peritumoral feeding arteries—can reliably identify the microvascular invasion (MTM) subtype of HCC preoperatively. By integrating these four features into a CEUS-clinical model, we have established a non-invasive preoperative prediction method for MTM-HCC. Notably, the incidence of MTM-HCC observed in our cohort (45 out of 239 cases, 18.8%) is very close to the rates reported in previous studies, such as Refs. [10,15,21], supporting the representativeness of our sample. These findings are consistent with the pathological hallmarks of MTM-HCC, which include thick macrotrabecular architecture, marked angiogenesis, and vessel-encapsulating tumor clusters [25,26]. Such features correspond to increased arterial supply and disorganized tumor vasculature, explaining the frequent presence of intratumoral and peritumoral arteries on CEUS, as reflected in prior imaging–pathology correlation studies [27]. Rapid tumor growth and vascular insufficiency may further lead to hypoxia and intratumoral necrosis, which has been previously described as a common imaging and pathologic correlate of aggressive MTM tumors [27]. Elevated AFP likely reflects the aggressive biological behavior and poor differentiation commonly observed in MTM-HCC [10,28].

Formally recognized by the World Health Organization in 2019, MTM-HCC has attracted growing research attention because its aggressive biological behavior is consistently linked to early recurrence and unfavorable survival outcomes [10,23,29]. From a preoperative standpoint, this creates a practical dilemma: although histopathology remains the diagnostic gold standard, therapeutic decisions often rely on imaging and laboratory data obtained before surgery. Consistent with previous reports [30], MTM-HCC patients in our cohort more frequently exhibited markedly elevated AFP levels, with nearly two-thirds (29 of 45) exceeding 1000 ng/mL, a range that in routine clinical practice often raises suspicion for biologically aggressive disease.

Higher AST levels were observed in patients with MTM-HCC, likely reflecting tumor-related hepatic injury associated with aggressive tumor growth rather than nonspecific inflammatory changes, as elevated AST has been linked to more extensive tumor necrosis and worse liver function in aggressive HCC subtypes [31]. Pathologically, MTM-HCC lesions exhibited poorer differentiation and increased proliferative activity, as indicated by consistently higher Edmondson–Steiner grades and elevated Ki-67 indices in MTM-HCC cases compared with non-MTM cases, supporting its hyperproliferative biology. These clinicopathological features are consistent with prior reports describing MTM-HCC as a biologically aggressive subtype characterized by rapid growth, invasive behavior, and a strong propensity for early dissemination [10].

The macrotrabecular architecture characteristic of MTM-HCC is frequently associated with hypoxia, necrosis, and vascular remodeling, all of which can further amplify inflammatory signaling within the tumor microenvironment. Although the biological mechanisms underlying MTM-HCC remain incompletely understood, accumulating evidence suggests that innate immune signaling may contribute to its aggressive behavior. Toll-like receptors (TLRs), particularly TLR4, are key mediators of hepatic inflammatory responses and have been implicated in liver injury, regeneration, and tumor development. Activation of the TLR4/NF-κB signaling pathway can promote hepatocyte proliferation while simultaneously amplifying pro-inflammatory cytokine production within the hepatic microenvironment [32]. Previous studies have shown that dysregulated TLR signaling is associated with tumor progression and immune modulation in hepatocellular carcinoma [33,34,35]. In the present study, patients with MTM-HCC tended to exhibit elevated inflammatory indicators, which may reflect a more active innate immune microenvironment. Although the precise relationship between TLR signaling and the MTM phenotype remains unclear, these findings raise the possibility that inflammation-related pathways contribute to the aggressive biological behavior of this subtype.

The vascular features identified in this study further reinforce current biological understanding of MTM-HCC. Angiogenesis has been repeatedly described as a defining hallmark of this subtype, with molecular studies highlighting upregulation of angiogenic pathways and overexpression of Ang-2 and VEGFA [24,25]. In parallel with these molecular observations, intratumoral arteries emerged as an independent predictor of MTM-HCC in our analysis, consistent with prior imaging-based reports [9,12,21]. From a structural perspective, the macrotrabecular architecture—defined by tumor trabeculae exceeding six cells in thickness and dominating the tumor mass—places substantial metabolic and perfusion demands on growing HCC. Such thickened trabeculae and associated architectural changes have been consistently described in MTM-HCC histology and are linked to aggressive tumor biology [24]. As malignant hepatocytes expand and replace normal parenchyma, the tumor increasingly relies on arterial blood supply, often resulting in arterialization and distortion of adjacent hepatic arterial branches and abnormal neovascular patterns to meet these demands. This vascular remodeling and increased arterial input likely underpin the frequent imaging observations of prominent intratumoral and peritumoral arterial features in MTM-HCC on contrast-based modalities [27]. In this setting, the presence of peritumoral feeding arteries, also identified as an independent predictor in our model, likely reflects this heightened vascular dependence. Such hypervascularity may partly explain the propensity of MTM-HCC to present as large, poorly encapsulated masses with a high risk of vascular invasion, recurrence, and metastasis [14,36,37].

Such tumor-driven vascular remodeling is expected to translate into distinct perfusion patterns on CEUS. Perfusion behavior on CEUS provided additional insight. In line with earlier investigations [21,22], the majority of MTM-HCC lesions in our cohort (40 of 45 cases) demonstrated hypoenhancement during the portal venous phase, frequently accompanied by pronounced washout. When the washout degree was stratified, significant washout was more commonly observed in MTM-HCC, suggesting altered tumor hemodynamics. The underlying pathophysiology likely reflects a combination of factors, including early venous drainage and a reduction in portal venous blood flow, which predispose lesions to relative hypoenhancement in the portal venous phase compared with background liver parenchyma [38,39]. In addition, rapid tumor proliferation and tightly packed cellularity reduce extracellular volume and limit intratumoral portal perfusion, contributing to more rapid and pronounced contrast washout [40]. Notably, unlike previous CT and MRI studies, in which MTM-HCC often appears as relatively hypoenhancing during the arterial phase, most lesions in our cohort showed arterial hyperenhancement on CEUS. This difference is most likely attributable to disparities in imaging principles and temporal resolution. CEUS allows real-time, continuous visualization of tumor perfusion and is particularly sensitive to early arterial inflow within abnormal microvasculature. MTM-HCC is characterized by active but disorganized angiogenesis, resulting in abundant yet inefficient arterial supply. Such transient high-perfusion states can be readily captured by CEUS, whereas CT and MRI, which rely on fixed arterial-phase acquisition, tend to depict later perfusion imbalance and intratumoral necrosis, leading to the appearance of arterial hypoenhancement. Therefore, arterial hyperenhancement observed on CEUS does not contradict prior imaging findings but instead highlights the complementary value of CEUS in depicting the dynamic vascular behavior of this aggressive HCC subtype [41].

Portal vein tumor thrombus (PVTT) was significantly associated with MTM-HCC in the univariate analysis (*p* < 0.001), but it did not retain statistical significance in the multivariable model (*p* = 0.961), suggesting that its predictive information may be captured by other variables such as intratumoral arteries and AFP. In this study, the MTM-HCC subtype was closely associated with the presence of PVTT. Macrovascular invasion, such as PVTT, is known to significantly worsen prognosis and increase the complexity of locoregional treatment decisions. Although Western guidelines have traditionally recommended non-surgical approaches for advanced PVTT, a growing body of evidence—particularly from Asian centers—supports the use of selective liver resection in well-defined patients with limited PVTT (confined to segmental or lobar branches) and preserved liver function, often achieving survival outcomes superior to those of non-surgical strategies [42,43]. Contemporary multimodality regimens—including resection with or without adjuvant therapy, TACE/TARE, stereotactic body radiotherapy, and systemic anti-angiogenic agents or immunotherapy—should therefore be tailored on an individual basis. Given the higher rate of venous invasion observed in MTM-HCC patients within our cohort, preoperative identification of this aggressive subtype, as proposed herein, may inform both surgical indications and perioperative planning in patients presenting with PVTT [44,45]. The potential clinical utility of our model was further supported by decision curve analysis, which demonstrated a sustained net benefit over a broad spectrum of threshold probabilities relevant to everyday practice (Figure 4). These findings suggest that the model could serve as a practical tool to pinpoint patients most likely to gain from aggressive preoperative planning, thereby aiding in individualized decision-making.

Artificial intelligence and image analysis techniques have unlocked new possibilities in the diagnosis and treatment of liver cancer. Recent progress in AI and quantitative imaging has reshaped how liver tumors are assessed non-invasively. Deep learning-based detection and segmentation algorithms have made it easier to identify lesions—particularly sub-centimeter nodules—while radiomics signatures extracted from ultrasound, contrast-enhanced ultrasound, CT, and MRI can capture intratumoral heterogeneity linked to molecular subtypes, microvascular invasion, and prognosis [46,47]. Furthermore, AI-driven pipelines can combine imaging data with clinical and lab results to generate personalized risk scores and predict treatment responses. This opens the door to integrating CEUS-based clinical models into automated decision-support systems. That said, before such tools can be adopted in routine clinical practice, several hurdles remain—chief among them being the need for standardized imaging protocols, rigorous external validation across devices and patient populations, and evidence from prospective studies [48,49].

Several limitations of this study merit consideration. The retrospective, single-center design may introduce unavoidable selection bias, and the limited sample size—particularly the small number of MTM-HCC cases—restricts the statistical power of subgroup analyses and precludes formal external validation. In addition, the AFP cutoff of 467 ng/mL was derived from ROC analysis within the study cohort; although this improved clinical interpretability, it reflects a data-driven choice that may limit generalizability. Therefore, these findings should be interpreted cautiously and require confirmation in larger, multicenter cohorts. In summary, MTM-HCC demonstrates distinct US and CEUS features that mirror its aggressive biological profile. By integrating intratumoral necrosis as well as intratumoral and peritumoral arterial features with serum AFP levels, we established a non-invasive model with good performance for preoperative identification of the MTM subtype. The observed imaging patterns are consistent with the pathological hallmarks of MTM-HCC, including dense macrotrabecular architecture and prominent angiogenesis. In conclusion, integrating CEUS features with clinical indicators demonstrates strong potential for the accurate, non-invasive preoperative identification of MTM-HCC.

Although the present study primarily addresses conventional hepatocellular carcinoma and the aggressive MTM-HCC subtype, it is important to recognize that a small number of uncommon primary hepatic malignancies can occasionally enter the differential diagnosis. Rare tumors such as primary squamous cell carcinoma of the liver have been reported [50,51], but they are typically identified only after thorough exclusion of an extrahepatic primary lesion and histopathological confirmation. Because their imaging manifestations are often nonspecific, distinguishing these entities from more common hepatic tumors based on imaging alone may be challenging. While such rare malignancies fall outside the scope of the current predictive model, acknowledging their existence provides broader clinical context and highlights the continuing importance of pathological evaluation. In situations where imaging findings and clinical or serological indicators are inconsistent, targeted biopsy may therefore remain an important step to achieve diagnostic clarification.

## 5. Conclusions

This study systematically evaluated the application value of conventional US and CEUS features combined with clinically relevant indicators in the preoperative identification of massive-trunk-mass hepatocellular carcinoma (MTM-HCC), a highly invasive histological subtype. A predictive model integrating imaging and clinical indicators was constructed. The results demonstrated that MTM-HCC exhibits hemodynamic and morphological features at the imaging level highly consistent with its biological characteristics, including intratumor necrotic areas, abundant intra- and peritumoural feeding arteries, and marked early enhancement. Concurrently, serum AFP levels were significantly elevated, correlating with AFP’s high proliferative activity and poor differentiation. By incorporating these key variables into multivariate analysis, a combined predictive model with favorable discriminatory and calibration capabilities was successfully established, providing an actionable radiological pathway for non-invasive identification of MTM-HCC.

## Figures and Tables

**Table 1:** Clinical and histopathological characteristics of patients in the two groups.

Characteristic	Non-MTM-HCC (*n* = 194)	MTM-HCC (*n* = 45)	*p*
Sex, *n* (%)			0.751
Female	43 (22.16)	9 (20.00)	
Male	151 (77.84)	36 (80.00)	
Age/year	57.60 ± 9.37	55.91 ± 10.57	0.288
AFP (ng/mL)	32.55 (4.29, 261.60)	1210.00 (475.00, 1210.00)	<0.001
CA199 (U/mL)	18.25 (9.19, 38.82)	23.90 (12.70, 45.10)	0.304
ALT (U/L)	41.00 (25.25, 114.75)	49.00 (33.00, 151.00)	0.152
AST (U/L)	56.50 (37.00, 140.50)	100.00 (53.00, 215.00)	0.005
ALB (g/L)	36.00 (31.90, 40.10)	36.70 (34.00, 38.10)	0.955
TBIL (μmol/L)	22.75 (16.15, 33.70)	25.40 (19.20, 34.50)	0.437
Hb (g/L)	143.00 (124.00, 156.50)	144.00 (119.00, 154.00)	0.828
PLT (×10^9^/L)	132.00 (88.25, 190.00)	152.00 (112.00, 192.00)	0.105
N (×10^9^/L)	4.16 (2.31, 6.72)	5.24 (3.49, 7.36)	0.045
L (×10^9^/L)	1.03 (0.70, 1.50)	0.84 (0.66, 1.32)	0.078
NLR	3.62 (2.16, 8.03)	5.19 (3.31, 14.00)	0.009
HBV, *n* (%)			0.677
No	46 (23.71)	12 (26.67)	
Yes	148 (76.29)	33 (73.33)	
Liver cirrhosis, *n* (%)			0.910
No	49 (25.26)	11 (24.44)	
Yes	145 (74.74)	34 (75.56)	
CD34, *n* (%)			0.577
-	16 (8.25)	2 (4.44)	
+	178 (91.75)	43 (95.56)	
Glypican3, *n* (%)			0.329
-	38 (19.59)	6 (13.33)	
+	156 (80.41)	39 (86.67)	
CK19, *n* (%)			0.014
-	161 (82.99)	30 (66.67)	
+	33 (17.01)	15 (33.33)	
Edmondson–Steiner grade (I-II/III-IV), *n* (%)			<0.001
I–II	172 (88.66)	30 (66.67)	
III–IV	22 (11.34)	15 (33.33)	
Ki67 (%)	30.00 (20.00, 40.00)	40.00 (30.00, 60.00)	<0.001

Unless otherwise specified, data are presented as median with interquartile range in parentheses. Abbreviations: MTM (thick trabecular-mass-type), HCC (hepatocellular carcinoma), AFP (alpha-fetoprotein), CA199 (carbohydrate antigen 199), ALT (alanine aminotransferase), AST (aspartate aminotransferase), ALB (albumin), TBIL (total bilirubin), Hb (hemoglobin), PLT (platelet), N (neutrophil), L (lymphocyte), NLR (neutrophil/lymphocyte ratio), HBV (hepatitis B virus). Data are presented as mean ± standard deviation.

**Table 2:** Results of comparison of US and CEUS features between the two groups of patients.

Characteristics	Non-MTM-HCC (*n* = 194)	MTM-HCC (*n* = 45)	*p*
Number of tumors, *n* (%)			0.133
Isolated	93 (47.94)	16 (35.56)	
Multiple	101 (52.06)	29 (64.44)	
Echo, *n* (%)			0.599
Hypoechoic	124 (63.92)	31 (68.89)	
Isoechoic	9 (4.64)	1 (2.22)	
Mixed echo	15 (7.73)	1 (2.22)	
Hyperechoic	46 (23.71)	12 (26.67)	
Maximum diameter (cm)	3.70 (2.20, 5.47)	6.60 (3.80, 8.20)	<0.001
Size classification, *n* (%)			<0.001
<3 cm	73 (37.63)	7 (15.56)	
2.3–5 cm	61 (31.44)	8 (17.78)	
>5 cm	60 (30.93)	30 (66.67)	
Echo homogeneity, *n* (%)			0.003
Uniform	53 (27.32)	3 (6.67)	
Non-uniform	141 (72.68)	42 (93.33)	
CDFI, *n* (%)			0.093
Grade 0	41 (21.13)	5 (11.11)	
Grade I	62 (31.96)	10 (22.22)	
Grade II	6 (3.09)	1 (2.22)	
Grade III	85 (43.81)	29 (64.44)	
Morphology, *n* (%)			0.267
Regular	149 (76.80)	31 (68.89)	
Irregular	45 (23.20)	14 (31.11)	
Tumor margin, *n* (%)			<0.001
Clear	111 (57.22)	11 (24.44)	
Indistinct	83 (42.78)	34 (75.56)	
Encapsulation, *n* (%)			0.070
Yes	98 (50.52)	16 (35.56)	
No	96 (49.48)	29 (64.44)	
Knot within a knot, *n* (%)			0.006
Yes	81 (41.75)	29 (64.44)	
No	113 (58.25)	16 (35.56)	
AP Start Enhancement Time (s)	15.00 (13.00, 18.00)	15.00 (14.00, 17.00)	0.820
Range Expanded After Enhancement, *n* (%)			0.045
Yes	37 (19.07)	3 (6.67)	
No	157 (80.93)	42 (93.33)	
AP enhancement pattern, *n* (%)			0.777
High enhancement	171 (88.14)	42 (93.33)	
Isotopic enhancement	20 (10.31)	3 (6.67)	
Low enhancement	3 (1.55)	0 (0.00)	
Arterial phase enhancement pattern, *n* (%)			<0.001
Synchronous enhancement	116 (59.79)	8 (17.78)	
Peripheral nodular enhancement	49 (25.26)	31 (68.89)	
Annular enhancement	11 (5.67)	2 (4.44)	
Centrifugal enhancement	18 (9.28)	4 (8.89)	
Start of decay time, *n* (%)			0.226
Ultra-early decay (<30 s)	8 (4.12)	4 (8.89)	
Early decay (<60 s)	68 (35.05)	20 (44.44)	
Late decay (>60 s)	118 (60.83)	21 (46.67)	
PVP decay degree, *n* (%)			0.027
No decay	56 (28.87)	5 (11.11)	
Mild decay	78 (40.21)	19 (42.22)	
Marked decay	60 (30.93)	21 (46.67)	
DP clearance degree, *n* (%)			0.127
No clearance	28 (14.43)	2 (4.44)	
Incomplete clearance	88 (45.36)	18 (40.00)	
Complete clearance	78 (40.21)	25 (55.56)	
No enhancement in necrotic areas, *n* (%)			<0.001
No	148 (76.29)	15 (33.33)	
Yes	46 (23.71)	30 (66.67)	
Intranodular artery, *n* (%)			<0.001
No	149 (76.80)	12 (26.67)	
Yes	45 (23.20)	33 (73.33)	
FA, *n* (%)			0.001
No	124 (63.92)	17 (37.78)	
Yes	70 (36.08)	28 (62.22)	
VTT, *n* (%)			<0.001
No	155 (79.90)	24 (53.33)	
Yes	39 (20.10)	21 (46.67)	
LI-RADS, *n* (%)			0.746
3	5 (3.38)	0 (0.00)	
4	30 (20.27)	5 (15.15)	
5	99 (66.89)	24 (72.73)	
M	14 (9.46)	4 (12.12)	

**Table 3:** Analysis of predictors in binary logistic regression for MTM-HCC.

Variable	*p*-Value of Univariate Analysis	*p*-Value of Multivariate Analysis	Multivariate OR (95%CI)
AFP ≥ 467 ng/mL	<0.001	<0.001	8.5 (4.2–17.30)
AST	0.575	0.334	1.00 (1.00–1.00)
N	0.656	0.509	0.97 (0.88–1.07)
NLR	0.128	0.054	1.06 (1.00–1.13)
Maximum tumor diameter	<0.001	0.529	0.93 (0.74–1.17)
Tumor size classification	<0.001	0.528	0.72 (0.27–1.97)
Echogenicity homogeneity	0.007	0.191	3.20 (0.56–18.29)
Tumor margins	<0.001	0.392	1.60 (0.54–4.71)
Double-peaked enhancement pattern	0.007	0.513	0.71 (0.26–1.97)
Enhanced area	0.056	0.178	3.09 (0.60–15.91)
Arterial phase enhancement pattern	0.011	0.684	0.90 (0.53–1.51)
Degree of PVP washout	0.009	0.896	0.95 (0.43–2.09)
Necrotic areas without enhancement	<0.001	0.003	5.92 (1.82–19.30)
Intramural arteries	<0.001	<0.001	6.61 (2.28–19.22)
FA	0.002	0.025	3.13 (1.15–8.50)
VTT	<0.001	0.961	1.03 (0.31–3.41)

Note: Data in parentheses are 95% confidence intervals. Abbreviations: MTM (thick trabecular-mass-type), HCC (hepatocellular carcinoma), AFP (alpha-fetoprotein), AST (aspartate aminotransferase), NLR (neutrophil/lymphocyte ratio), PVP (portal venous phase < 120 s), FA (peritumoral feeding artery), VTT (venous tumor thrombus), OR (odds ratio), CI (confidence interval).

**Table 4:** Confusion matrix.

AUC (95% CI)	Accuracy (95% CI)	Sensitivity (95% CI)	Specificity (95% CI)	PPV (95% CI)	NPV (95% CI)	Cut Off
0.915 (0.860–0.970)	0.879 (0.830–0.917)	0.887 (0.842–0.931)	0.844 (0.739–0.950)	0.961 (0.932–0.989)	0.633 (0.511–0.755)	0.255

Note: AUC (area under the curve), Accuracy (accuracy), Sensitivity (sensitivity), Specificity (specificity), PPV (positive predictive value), NPV (negative predictive value), Cut-Off (critical value).

## Data Availability

The datasets and materials supporting the findings of this study are available from the corresponding author upon reasonable request. Patient-sensitive data will be anonymized prior to sharing to protect privacy.
